# “What bothers the heart”: a phenomenological study of adolescents’ experiences and interpretations of school-based gender-based violence in Kinshasa, DRC

**DOI:** 10.1080/26410397.2026.2702756

**Published:** 2026-07-13

**Authors:** Julie H. Hernandez, Muanda F. Mbadu, Lorentz Murrairi

**Affiliations:** a Associate Professor, Celia Scott Weatherhead School of Public Health and Tropical Medicine, Tulane University, New Orleans, LA, USA.; b Director, Programme National de Santé des Adolescents, Kinshasa, Democratic Republic of Congo; c M&E Expert, PNSA, Kinshasa, Democratic Republic of Congo

**Keywords:** school-based gender-based violence, adolescent health, adolescent rights, Democratic Republic of Congo, phenomenological qualitative research

## Abstract

Gender-based violence in high schools undermines adolescents’ rights, well-being and opportunities at a critical moment in their psycho-social development. While violence prevalence has been partially assessed in sub-Saharan Africa, there remain gaps in understanding how adolescents themselves define, interpret and navigate these situations. This phenomenological qualitative study conducted in four high schools in Kinshasa (DR Congo) centres adolescent voices to explore how they perceive, legitimise, and manage school-based gender-based violence. It aims to identify the individual beliefs and practices that can be used to interpret, accept or challenge violence, as well as the institutional structures that produce and legitimise these conditions. In December 2024, 90 adolescents aged 15–17 participated in focus group discussions and individual interviews. Data were thematically analysed using NVivo and iterative validation. Violence was pervasive and multifaceted, encompassing physical abuse, humiliations, economic coercion, and sexual harassment. Some instances of violence were seen as legitimate based on institutional and gendered expectations, and victim blaming was frequent. Community judgement deeply framed students’ narratives, leading to stronger condemnation of arbitrary acts, public humiliations, and economic sanctions. Correspondingly, fear of reputational harm discouraged reporting, and silence and conformity emerged as key coping strategies. Students’ ability to respond effectively appeared entirely constrained by their pre-existing reputation in the community, which determined whether they were believed, supported, or blamed. Findings indicate that addressing GBV in Kinshasa high schools requires both community- and school-level interventions that confront the norms legitimising violence and victim-blaming, policies that strengthen accountability mechanisms and protect students who report abuse, and further research on adolescent-led strategies to transform harmful institutional and social expectations. Supporting adolescents’ voices in defining and resisting school-based GBV is critical to safeguarding their rights and fostering equitable education. *DOI: 10.1080/26410397.2026.2702756*

## Plain language summary

This study looks at how adolescents in Kinshasa, DR Congo, perceive, understand and respond to violence in their schools. Gender-based violence includes physical, verbal, economic, and sexual abuse. It violates young people’s rights and decreases their chances to succeed in school and to lead healthy, successful lives. Yet little is known about how students themselves experience and cope with such violence on a daily basis. The goal of this study is to examine: (1) how adolescents make sense of violence in their daily lives; (2) how they decide whether to accept or challenge it; (3) how schools and other institutions help maintain these patterns of violence. We spoke with 90 high school students aged 15–17 through group discussions and interviews in December 2024. We asked students “what bothers them” at school, allowing them to describe their experiences in their own words. Students reported many forms of violence, from slaps and insults to teachers demanding sex in exchange for grades. Sometimes the students considered some types of violence acceptable. They thought their reputation in the community affected how other people reacted to their suffering. Victims seen as “respectable” were more likely to be believed and supported, while others were blamed. Many students preferred to stay silent rather than report abuse, for fear of being shamed or punished further. They also felt that school personnel protected each other rather than supporting victims. The results show that ending gender-based violence in Kinshasa needs schools and communities to work together to change unfair norms, make abusers face consequences, and help students speak up. Supporting adolescents who speak out and rejecting the idea that violence is normal is key to making schools safer and more just.

## Résumé

Les violences sexistes dans les écoles secondaires compromettent les droits, le bien-être et les perspectives des adolescents à un moment crucial de leur développement psychosocial. Si la prévalence de ces violences a été partiellement évaluée en Afrique subsaharienne, il subsiste des lacunes quant à la manière dont les adolescents eux-mêmes définissent, interprètent et affrontent ces situations. Cette étude qualitative phénoménologique, menée dans quatre écoles secondaires de Kinshasa (République démocratique du Congo), donne la parole aux adolescents pour explorer comment ils perçoivent, légitiment et gèrent les violences sexistes en milieu scolaire. Elle vise à identifier les croyances et pratiques individuelles qui peuvent servir à interpréter, accepter ou contester ces violences, ainsi que les structures institutionnelles qui les produisent et les légitiment. En décembre 2024, 90 adolescents âgés de 15 à 17 ans ont participé à des discussions de groupe et à des entretiens individuels. Les données ont été analysées thématiquement à l’aide du logiciel NVivo et par validation itérative. Les violences étaient omniprésentes et avaient de multiples facettes, englobant les mauvais traitements physiques, les humiliations, la coercition économique et le harcèlement sexuel. Certains actes de violence étaient perçus comme légitimes au regard des attentes institutionnelles et sexospécifiques, et les victimes étaient fréquemment stigmatisées. Le jugement de la communauté influençait profondément les récits des élèves, entraînant une condamnation plus ferme des actes arbitraires, des humiliations publiques et des sanctions économiques. Parallèlement, la crainte de nuire à leur réputation décourageait les élèves de signaler les faits, et le silence et la conformité sont apparus comme les principales stratégies d’adaptation. La capacité des élèves à réagir efficacement semblait entièrement limitée par leur réputation préexistante au sein de la communauté, qui déterminait s’ils étaient crus, soutenus ou blâmés. Les résultats indiquent que la lutte contre les violences sexistes dans les écoles secondaire de Kinshasa exige des interventions, tant au niveau communautaire que scolaires s’attaquant aux normes qui légitiment la violence et jettent le blâme sur la victime, des politiques renforçant les mécanismes de redevabilité et protégeant les élèves qui signalent les abus, et des recherches complémentaires sur les stratégies menées par les adolescents pour transformer les attentes institutionnelles et sociales néfastes. Il est essentiel de soutenir la parole des adolescents pour définir et combattre les violences sexistes en milieu scolaire afin de protéger leurs droits et de promouvoir un enseignement équitable.

## Resumen

La violencia basada en género en las escuelas secundarias (preparatorias) socava los derechos, el bienestar y las oportunidades de las adolescentes en un momento crítico en su desarrollo psicosocial. Si bien la prevalencia de violencia ha sido evaluada parcialmente en África subsahariana, aún existen lagunas en entender cómo las adolescentes definen, interpretan y navegan estas situaciones. Este estudio cualitativo fenomenológico realizado en cuatro secundarias de Kinshasa (RD del Congo) centra las voces de adolescentes para explorar cómo perciben, legitiman y manejan la Violencia Basada en Género en las escuelas. El objetivo del estudio es identificar las creencias y prácticas individuales que pueden utilizarse para interpretar, aceptar o cuestionar la violencia, así como las estructuras institucionales que producen y legitiman estas condiciones. En diciembre de 2024, 90 adolescentes de 15 a 17 años participaron en grupos focales y entrevistas individuales. Se analizaron los datos temáticamente utilizando NVivo y validación iterativa. La violencia era generalizada y multifacética, y consistía en maltrato físico, humillaciones, coerción económica y acoso sexual. Algunos incidentes de violencia fueron considerados como legítimos según las expectativas institucionales y de género, y con frecuencia se culpaba a las víctimas. Las narrativas de las estudiantes eran juzgadas profundamente por la comunidad, lo que propiciaba mayor condena de actos arbitrarios, humillaciones públicas y sanciones económicas. En la misma medida, el temor a sufrir daños a la reputación disuadía a las adolescentes de denunciar la violencia, y el silencio y la conformidad surgieron como estrategias clave para afrontar la situación. La capacidad de las estudiantes para responder de manera eficaz parecía estar totalmente limitada por su reputación preexistente en la comunidad, que determinaba si se les creía, se les apoyaba o se les culpaba. Los hallazgos indican que para abordar la VBG en las secundarias de Kinshasa se necesitan intervenciones comunitarias y escolares que confronten las normas que legitiman la violencia y la culpabilización de las víctimas, políticas que refuercen los mecanismos de rendición de cuentas y protejan a las estudiantes que denuncien maltrato, y más investigaciones sobre estrategias lideradas por adolescentes para transformar las expectativas institucionales y sociales dañinas. Apoyar las voces de las adolescentes para definir y resistir a la VBG en las escuelas es fundamental para salvaguardar sus derechos y fomentar una educación equitativa.

## Introduction

Adolescence is a critical stage for physical, emotional, and social development, particularly in relation to sexual and reproductive health. In the Democratic Republic of Congo (DRC), an estimated 25 million of the country's more than 110 million inhabitants are between the ages of 10 and 19. For many, this pivotal period unfolds in environments that expose them to considerable risks, with potentially lasting consequences for their long-term reproductive, but also socio-economic and psychological trajectories. Available research on gender-based violence (GBV) in the country suggests that nearly a quarter (22%) of women have experienced at least one instance of sexual violence before the age of 18.^1^ The Demographic and Health Survey (DHS) 2023–2024 estimates that one in five girls (20%) aged 15–19 has already been pregnant,^2^ raising strong concerns given that adolescent pregnancy carries elevated health risks and is associated with long-term socioeconomic disadvantage.^3–7^

Education can play a protective role and is crucial to poverty alleviation by strengthening adolescent girls’ autonomy and agency in making informed decisions about their lives, health, and futures. It is also instrumental to empowering adolescents and fostering gender equality by shaping gender norms, expectations, and life trajectories.^8^ But while primary education enrolment and completion rates (86% for boys and 79% for girls) have increased in the DRC following the implementation of free education policies in 2019, a severe drop-off persists at the secondary level, along with an increase in the gender gap. UNESCO country estimates show gross enrolment in high schools to be 70% for boys (with a 64% completion rate) but only 44% for girls (with a 51% completion rate).^6,7^ Factors limiting adolescent and particularly girls’ access to secondary education in sub-Saharan Africa, including unequal domestic responsibilities, safety concerns, inadequate access to menstrual hygiene supplies and separated bathrooms, early marriage and childbearing, have been well-researched.^9^ Underlying economic concerns linked to the tuition, uniforms and other schooling costs almost systematically compound those issues and secondary education often represents significant opportunity costs and financial sacrifices for families.^10,11^

The limited data available for the DRC suggest that educational settings themselves might be a locus of increased risk of gender-based and sexual violence for youth and adolescents. Very few studies have been conducted to estimate the extent of school-based gender-based violence in the country, but in the DHS 2013–2014, nearly one in ten women (9.6%) reported physical or sexual violence perpetrated by a teacher.^12^ This figure could be seriously underreported, as a study conducted by UNICEF in 2014 indicated that about 46% of Congolese schoolgirls had been victims of sexual harassment, abuse and violence committed by their teachers or other school personnel.^11^ A more detailed study conducted in neighbouring Republic of the Congo also suggested that 30–40% of high school students had been victims of physical, economic or sexual violence and 73% had faced verbal or psychological violence.^13^ Earlier research conducted in Ghana, Kenya, and Mozambique also highlighted the prevalence of physical punishment in educational settings, as well as sexual violence perpetrated by peers and teachers.^14–16^

School-based gender-based violence encompasses a range of acts, from verbal harassment and physical punishment to coercive sexual behaviour, perpetrated within educational settings by peers, teachers and school personnel and shaped by the power hierarchies existing in these environments. Although there are no reliable estimates of school dropout directly attributable to violence in DRC, evidence from other sub-Saharan African contexts suggests a strong association between the two phenomena: in Ghana, exposure to bullying and corporal punishment has been linked to significantly higher odds of absenteeism and dropout,^17^ and in South Africa, nearly one in five learners experiencing repeated school violence reported considering leaving school early.^18^ Globally, evidence suggests that such violence can lead to lower educational attainment, increased school dropout rates, and early or forced sexual debut, all of which have profound effects on future sexual and reproductive health outcomes.^19,20^

Crucially, school-based GBV also constitutes a violation of fundamental human rights, including the right to education, but also the rights to bodily autonomy and dignity. While the right to education has been more frequently addressed through programmatic and policy efforts to increase school enrolment and completion (in line with the Sustainable Development Goals), the other two remain comparatively under-studied, in part because violations of autonomy and dignity are more difficult to operationalise and measure across settings. Yet these dimensions are central to adolescents’ lived experiences of harm and shape whether schooling is not only accessible, but safe, equitable, and respectful.

In the DRC, the limited epidemiological literature on school-based sexual and gender-based violence has primarily focused on quantifying incidence through retrospective surveys and teacher-reported data, relying on pre-categorised acts of violence (e.g. slapping or beating, inappropriate touching or forced sexual intercourse).^21^ To date, however, no research has examined adolescents’ own experiences and interpretations of what they themselves define as violence in school environments, particularly with respect to perceived violations of autonomy and dignity. This limits both accurate estimation and effective prevention efforts by overlooking forms of harm adolescents recognise as a violation and by neglecting how such harm is lived and navigated in practice. Responding to prior recommendations to explore how children conceptualise violence in order to adequately measure and address it,^22^ this study adopts a phenomenological approach that centres adolescent voices in the definition, interpretation, and navigation of everyday violence in Kinshasa’s school settings.

By grounding the analysis in adolescents’ lived experience, this research centres them as rights-holders and active interpreters of harm, rather than passive recipients of violence. It seeks to identify the individual beliefs and practices that can be used to interpret, accept or challenge violence,^23^ as well as the institutional structures that enable and legitimise violations of autonomy and dignity within school settings. Its findings aim to inform inclusive and rights-based policy and programing in fragile settings.

## Methods

This qualitative study was conducted over a 12-day period (2–14 December, 2024), in four public and private high schools located in four Educational Provinces (PROVED) of Kinshasa (Lukunga, Tshangu, Funa and Mont Amba). The purposive sample was designed to capture both the geographic diversity of Kinshasa as well as variations in school governance and affiliation, including fully public, non-religious private, Protestant and Catholic, which represent the most common institutional types in Kinshasa. The target population included adolescents aged 15–17 attending the selected schools. Inclusion criteria for both focus group discussion (FGD) and in-depth interview (IDI) were similar: participants had to be between the ages of 15 and 17, speak French or Lingala, have been enrolled at the school for a minimum of two years and consent to participate in the study. In line with the study’s phenomenological aims, participant selection emphasised diversity of school experiences and perspectives over statistical representativeness.

The data collection team was composed of six personnel from the National Adolescent Health Program (PNSA) with extensive experience in qualitative data collection. All were less than thirty years old and had attended high school in Kinshasa, although not in the establishments selected for the study. Data collectors received a three-day training, including refresher techniques for qualitative methods, practical exercises and role-playing, ethical issues and best practices for youth-centered research. The training was also the occasion to complete a first review and discussion of the FGD and interview guides. It was followed by a pre-test conducted with two focus groups (six participants per group) and five individual interviews with students from schools not included in the research sample. Results from the pre-test were discussed with the entire team and youth interns working with PNSA to further inform data collection guides.

The approach in developing and refining the FGD and interview guidelines was to use a layered thematic inquiry beginning with broad and non-sensitive questions on boys’ and girls’ experiences in school before gradually narrowing the discussion towards more specific issues such as sexual violence. This progressive structure helped to develop a rapport with the participants and build their readiness to move into more difficult topics. It also provided rich contextual data that informed the definition of key concepts for this study and enabled participants to introduce themes organically, while still allowing the research team to converge on key topics through sequenced questions. In order to prioritise adolescents’ own language and interpretations, moderators and interviewers were specifically trained not to offer pre-defined categorisation of violence, but to let the participants both define and qualify what constituted violence in their mind. This is consistent with a rights-based approach that recognises adolescents as knowledgeable agents rather than passive recipients of protection.

This was especially important as, in Lingala, the concept of “violence” does not have a single direct equivalent and is typically expressed through descriptive terms that vary depending on the type and severity of the act. Physical violence is often described with verbs such as *kobéta* (to hit), while psychological or verbal violence may be conveyed through terms like *kobangisa* (to intimidate) or *koloba mabe* (to speak badly). Sexual violence is commonly referred to through euphemisms such as *ko zua muasi to mobali na makasi* (to forcefully use someone). Since all these wordings would lead the conversations towards pre-categorised acts of violence, an agreement was reached during the training and pre-test phases to first simply ask for examples of “what bothers you?” (“*Eloko nini ezo tungisa motema na bino”* in Lingala*, “ce qui vous dérange le coeur”* in French, or literally *“something that bothers your heart”* as a first proxy for violence). FGD and interview participants later spontaneously expanded this basic definition by describing “violent” acts and behaviours as “what is not normal”, “what I don’t like”, “what is weird”, “what is illegal or forbidden by law”, and “what is against nature”.

The study objective and methods were initially broadly presented to both the school personnel and students, with the initial presentation mentioning discussions of school environments, learning conditions and gender equality. Students aged 15–17 at the selected schools who volunteered to participate in the study were then individually consented by the research team. The researchers broadly explained that the research specifically concerned school- and gender-based violence and that students could choose between participating in FGDs or individual interviews. Students who agreed to participate in FGDs were then invited to join a small group of their peers at a set date and in a neutral location (outside of school). FGDs were limited to six people per group to decrease the chances of a breach of privacy or confidentiality, and groups were assembled to ensure that participants did not know each other well. All FGDs were segregated by gender, with moderators and note takers of the same gender as participants. At the beginning and throughout the discussion, moderators reminded students not to use identifiable names and to remain respectful of their fellow participants’ opinions. FGDs lasted between 90 and 120 minutes and were all audio-recorded after the participants gave their permission.

Students who preferred to participate in individual interviews (IDI) were invited to meet with a data collector at a time and location of their preference, and each participant also completed the written informed consent process. Individual interviews lasted between 60 and 90 minutes and were also audio-recorded with the participants’ permission.

All interviews and focus group discussions were audio-recorded and transcribed verbatim. As most transcripts included a mix of Lingala and French, two separate teams at PNSA translated them fully into French, using cross-validation techniques where the Lingala translation was uncertain. As all members of the research team, including the corresponding author for this paper, are native French speakers, the analysis was completed using the French transcripts. All direct quotes presented in this paper were translated by the corresponding author, who has been a public health researcher in the U.S. for over 20 years and has conducted extensive field programmatic work, as well as quantitative and qualitative research on sexual and reproductive health and rights in the DRC since 2010. The senior authors for this study, a French female researcher and a male Congolese programmatic director, have worked closely together on similar research efforts since 2014 and have a joint record as strong advocates of sexual and reproductive health and rights (SRHR) for youths and adolescents in scientific and policy efforts in country. However, we acknowledge that our cultural background and professional lenses might have biased some of our analysis and made every effort to involve a large and diverse team in the classification and interpretation of the findings. Furthermore, as none of the authors are native English speakers, we have used some generative AI (ChatGPT) to occasionally suggest alternative phrasing or tighten some of the language. The research design, protocol development, data collection and analysis, and initial drafting of the full manuscript were however entirely and independently done by the authors.

The thematic analysis used NVivo software to systematically code and organise the data. The three authors completed the coding independently and later compared their results to increase reliability and consistency. The initial coding framework was developed based on the interview guides, then all identified themes and interpretations were iteratively refined through discussions with the whole research team, including FGD and IDI moderators and young PNSA interns. Coding discrepancies were resolved through consensus and recurrent themes were continuously validated across datasets.

While it was not possible to share the findings from the study with the participants themselves due to conflicts in the school calendar, results from this study have been presented to key stakeholders in Kinshasa, including personnel from the Ministry of Education, the school districts, and the schools themselves, NGOs working in the field of adolescent SRHR and empowerment, and youth organisations, including the International Youth Alliance for Family Planning in République Démocratique du Congo (IYAFP RDC).

Older adolescents (aged 15 and above) in the DRC are almost systematically considered emancipated adults in population-based surveys. Considering the sensitive nature of the topics discussed and the added risk to the students if other adults knew of their participation, written informed consent was collected directly from the participants before all data collection procedures started.

Ethical approval for this study was provided by the Ethics Committee for the Kinshasa School of Public Health (ESP/CE/120B/2024, approved Nov 4, 2024) and the Institutional Review Board at Tulane University Celia Scott Weatherhead School of Public Health and Tropical Medicine (# 2024-1815-Non-Tulane-Site, approved Nov 19, 2024)

## Findings

In total, 90 adolescents aged 15–17 were included in the study, with 36 (24 girls and 12 boys) participating in FGDs and 54 (36 girls and 18 boys) participating in IDIs (Table 1).

**Table 1.** Characteristics of participants

Number of participants	FGD	IDI	Total
**Female**	**24**	**36**	**60**
Private school	6	9	15
Public school	6	9	15
Religious school – Catholic	6	9	15
Religious school – Protestant	6	9	15
**Male**	**12**	**18**	**30**
Private school		4	4
Public school		4	4
Religious school – Catholic	6	5	11
Religious school – Protestant	6	5	11
Total	36	54	90

The findings below are organised around three interrelated themes that emerged inductively from our phenomenological analysis: adolescents’ experiences of violence, their interpretations of its meaning and legitimacy, and the strategies they deploy to navigate or respond to it. Three subthemes were identified in the first section: (1) the integration of routine physical violence and everyday forms of humiliation in adolescents’ schooling experiences, (2) economic coercion and abuses of power also being defined and experienced as violence, and (3) the pervasiveness of sexual misconduct from peers and teachers and “sex-for-grades” blackmail practices. Adolescents’ understanding of violence was organised around five subthemes: (1) violence’s definition through consent and relational context, (2) framing violence from teachers as a pedagogical necessity, (3) gender expectations and victim-blaming logics, (4) perceptions of arbitrary violence and betrayal of teachers’ duty of care, and (5) social reputation as a mediator of blame. Finally, available options to navigate or address violence were also organised around five sub themes: (1) silence and avoidance as primary coping strategies, (2) mistrust in institutions and anticipated injustice, (3) internalisation of responsibility and behavioural self-regulation, (4) perceived unfairness of gendered asymmetries in available coping strategies, and (5) moral standing, credibility and the threat of compounded reputational harm.

### Experiencing violence

Both focus group discussions (FGDs) and individual interviews (IDIs) revealed a broad and diverse range of behaviours categorised as “violent” (“what bothers you”) by participants.

#### Routine physical violence and everyday forms of humiliation

Physical violence appeared omnipresent in students’ school lives. This included routine disciplinary practices such as teachers and staff slapping or shoving students, despite the formal prohibition of corporal punishment under Congolese law,* as well as fights among classmates and students physically confronting teachers. While all students reported being slapped (“*chicoter*”) as a common disciplinary measure, a majority, however, appeared more affected by insults and public humiliations, particularly those targeting their families. Losing face in front of the class was identified more often and more spontaneously as a painful event than being beaten.

“*The teachers giving you discipline is one thing but often they insult you, if you don’t know the answer, instead of explaining or encouraging you, they say ‘you’re stupid, you’ll be unemployed like your parents, you’re like dogs I cannot teach you anything’. And when they say that in front of your classmates, it breaks your heart and then you cannot concentrate anymore*.” [Boy IDI-M-17-037]

#### Economic coercion and abuses of power

In the context of widespread poverty in the Democratic Republic of Congo, students also assimilated certain punishments as a form of economic violence. Common examples included being asked to bring chalk for the class, purchase a teacher’s lunch, or contribute money for school repairs, such as bricks to fix the building. Teachers tearing up notebooks over poorly done assignments were perceived as particularly painful as it was both humiliating and engendered additional costs for the students and their families.

“*The teacher will tear your notebook because your writing is poor, and it means your parents have to buy a new one and that gets you in trouble at home too*.” [Girl FGD-F-17-058]

“*Sometimes you do nothing wrong, and a teacher searches your bag and confiscates your phone and then you have to pay him XOF 5,000 [about $1.8] to get it back, they don’t even care if your family is poor, it’s very unfair*.” [Boy FGD-M-15-088]

#### Pervasiveness of sexual misconduct from peers and teachers, and “sex-for-grades” blackmail

When asked more specifically about instances they would consider sexual violence, students again displayed a relatively broad understanding of the concept. While few respondents mentioned instances of rape in school settings, nearly all identified lower-level acts such as fondling and harassment as frequent forms of violence. Most respondents also described instances of physical, verbal or online pressure for sexual favours, if not outright sexual relationships, and categorised them as violent. These included classmates and teachers touching girls’ breasts and buttocks during class or recess but also spreading rumours and shunning girls who do not agree to provide sexual favours.

“*There’s a [male] teacher who always says, ‘the first one I catch cheating in the exam, I will punish them and if it’s a girl, I’ll kiss her and I’ll squeeze her breasts’*.” [IDI-F-15-002]

By far, the most frequently mentioned and disruptive form of violence was teachers blackmailing students for sexual favours in exchange for grades. Although this dynamic primarily involved male teachers targeting female students, a minority of respondents also acknowledged cases of female teachers harassing male students.

“*With teachers this sometimes happens when you have difficulty understanding certain lessons, then the teacher can ask you to have sex with him so that he gives you the points, if the girl is weak and she is also afraid to repeat the class, she can also accept the teacher's request to have sex with him […] Sometimes you can also be smart in his class but when he calls you into their office to buy him things (water, juice etc.) and if you don’t have the money, he will also take advantage to ask you to go out with him*.” [Girl IDI-F-16-011]

“*Sometimes even female teachers, they will ask boys to stay after class because they want to kiss them and touch them. And they will promise extra points in the class if the boy lets them do what they want and even boys are not always comfortable with that*.” [Boy FGD-M-17-080]

Harassment could also go beyond school settings with several respondents stating that they had been directly contacted by teachers on WhatsApp or other social media apps, with requests to meet or to send explicit pictures.

“*Once on WhatsApp I see a number I don’t recognize, and someone says ‘Hi’ and then ‘Send me nudes!’ And I realized it was our Head Teacher, and I didn’t know what to do. I received so many messages from him asking for sexy pictures*.” [Girl FGD-F-17-063]

All participants reported that those who refused sexual advances faced serious academic consequences, ranging from being ignored in class to receiving bad grades or even expulsion. This made any student coming from a lower economic background vulnerable to sexual coercion as the threat to fail and repeat a year would have serious financial implications for their family and their continued education.

“*The director of our school let me get in class even though I couldn’t pay the tuition that semester. But then he said we should have sex now and I refused, so he fired me. I had to wait to find the money to continue going to school*.” [Girl IDI-F-15-002]

#### Understanding violence

##### Defining violence through consent and relational context

Students in focus groups and individual interviews displayed a complex understanding of the legitimacy of what they experienced as violence, ranging from sophisticated definitions of the notion of “consent” to allowing grey zones in allocating responsibilities for physical, verbal and sexual abuse. Respondents for example clearly differentiated between romantic relationships between students, which, despite being prohibited by school regulations, “*happened all the time*”, and situations deemed “*weird*”, “*not normal*” or “*illegal*”. Furthermore, the majority of participants were able to isolate the notion of consent from the acts themselves, as suggested by their acknowledgement of the possibility of violence even within established relationships:

“*I would say yes, sexual violence can happen to people who are also in a relationship, this is the case in our relationships with our boyfriends, it sometimes happens that the man starts to force you to make love even if you don't want to do it, this is already sexual violence*.” [Girl IDI-F-16-019]

##### Violence from teachers framed as pedagogical necessity

Despite this theoretical understanding of the notion of consent, two factors seemed to influence students’ assessment of the acts of violence they witnessed or experienced at school: their perceived legitimacy and the pre-existing reputation of the victim. In fact, most students indicated that physical punishment, including slapping or beating a student, was completely justified if the student was “*insolent*”, “*disrespectful*” or “*disregarded the school rules*”. Teachers in this situation were perceived as fulfilling their institutional responsibilities as educators.

“*The fact that some students protest against the teachers’ decisions, if a teacher hits him, and then the student continues to protest or hits his teacher back, this is what increases the case of physical violence at school. I personally find that it is normal for a teacher to whip a student as part of educating him or to discipline him to teach a lesson to other students who are disturbing the class*.” [Boy FGD-M-15-079]

##### Gender expectations and victim blaming logics

This acceptance of certain forms of violence expanded into victim blaming with both genders being perceived as displaying behaviours that attracted abuse. For boys, punishment was seen as legitimate because they were perceived as inherently “*stubborn*” and “*disruptive*”. Similarly, the majority of respondents assigned some blame to the behaviours of the girls themselves, principally through the way they dressed and acted in class.

“*There are other girls in our school who want to be seen at all times, girls who come to school only to look for boys, they want to be noticed by everyone at school at all costs, they wear short skirts, tight clothes, these kinds of girls if they suffer from violence, I think it's their fault*.” [Girl IDI-F-15-027]

“*The agents of the Ministry of Public Health told us that it is not at all good to wear transparent clothes if we want to prevent men/boys from falling into sexual violence […] I know a man who raped a girl because of her clothing [That is why] I would ask girls to avoid clothes that are too sexy because it also attracts men/boys and forces them to have negative thoughts about them, or to have sexual desires at all costs*.” [Girl FGD-F-17-078]

“*It can be her fault if she doesn’t respect herself, because there are also boys who are afraid to approach girls who respect themselves, but those who don’t respect themselves experience these kinds of trouble*.” [Boy FGD-M-17-084]

In the examples above, gendered and institutional expectations contributed to normalise and legitimise violence along three key assumptions: violence is acceptable if it supports an educational mission; men’s sexual urges cannot be controlled and must be satisfied; and it is women’s responsibility not to attract their attention.

##### Arbitrary violence and betrayal of teachers’ duty of care

At the opposite end of the spectrum were acts students saw as entirely arbitrary and categorised as the worst form of violence they or their classmates could experience. Participants universally condemned instances where teachers and staff randomly victimise students, as well as when punishments appeared disproportionate to the infraction (e.g. arriving five minutes late, wearing the wrong colour shoes, not wearing your hair short enough). Female respondents in particular dreaded being singled out by a teacher’s attentions.

“*Once in computer class, the teacher turned around and slapped me, and I didn’t even know why. He said, ‘you were talking too much’ but I said I was not and then he slapped me again and said, ‘that’s because your shirt is not buttoned correctly’. I’m always afraid of that teacher*.” [Girl IDI-F-15-018]

“*It’s the worst thing that can happen is when you’re sitting quietly and you’re not insolent, but then a teacher falls in love with you, and they want to touch you*.” [Girl FGD-F-15-065]

This kind of transgression was all the more highlighted by students as they perceived it as being an institutional dereliction of duty and “*almost against nature*”. Schools were described as “*almost like a family*” and teachers “*should act like protective parents*”.

“*When you’re at school, you’re still a child, you’re not dressing like a woman, and if a teacher wants to go out with you, it’s wrong. Not just because you’re 15 and maybe they’re 50, but because they should be like a father to you. And that’s sexual violence*.” [Girl FGD-F-15-066]

In the example above, teachers harassing students who “*have done nothing*” are condemned more severely because they are perceived as transgressing their educational mission and betraying students’ expectations.

##### Social reputation as a mediator of blame

Besides the perceived arbitrariness and unfairness of certain situations however, the transgressive nature of violence was also mediated in adolescents’ statements by the pre-existing reputation of the victims.

“*It depends on your behavior before […] if people know you’re well-behaved, if you dress correctly, if you’re smart and people harass you, then they will blame the perpetrator of the violence and not you*.” [Girl FGD-F-15-074]

“*For people in the community if you are an insolent child, they may say that it is normal that negative consequences happen to you, but if you are an intelligent, serious child who also respects people, they will feel sorry for you*.” [Boy IDI-M-16-044]

Overall, acts of violence were therefore almost never understood by students as independent occurrences, but always assessed based on institutional, gender and contextual expectations, which have important consequences on the strategies open to victims for navigating these situations.

#### Navigating violence

##### Silence and avoidance as primary coping strategies

As violence was both widespread and multifaceted in students’ school experience, with some instances being partially legitimised by the victims’ behaviours, the majority of students appeared largely defenceless. Both the FGD and interview transcripts almost solely mentioned avoidance strategies to limit one’s exposure to this kind of situation. When asked, ‘What advice would you give to a friend in this situation?’ participants most frequently recommended remaining silent, forgetting the incident, and avoiding any action that could further damage their reputation, compounding the harm they had already endured.

“*I would tell them to remain silent or pretend not to hear anything in such situations […] they cannot tell this to their friends because [the friends] can also betray them by going to tell everything to the perpetrator of this violence and this can spread to the whole school, which is not good at all either*.” [Girl FGD-F-15-066]

“*Some advice I would give to this person who has suffered this kind of violence is, do not think too much about it, because it will always make you feel guilty, what is past is already past, you just have to look forward*.” [Girl IDI-F-16-019]

##### Mistrust in institutions and anticipated injustice

The main justification for remaining silent is a lack of trust in the educational and legal institutions. Overall, students exhibited a deeply entrenched lack of faith in the adults in their community and the expected rule of justice. When asked “what could happen if someone reports a teacher for harassment”, most responses were organised as “maybe … but maybe … ” statements, with a large swath of uncertainty regarding the enforcement of existing norms and regulations.

“*Most of the time, these are situations where you can't say anything or do anything, this is the case of a teacher who wants to go out with me at all costs, who bothers me at any time and asks for my phone number, as a student I don't want to say anything or I don't want to report it, because sometimes when you report such a case to the management and the teacher denies everything, due to lack of evidence the student can be permanently excluded from the school. As we say ‘wolves don’t eat each other’ so the director is not going to be on my side. That’s why I can't do anything or react; I just want to let this problem go*.” [Girl IDI-F-17-024]

While several students suggested “going to the police” would be important to prevent recidivism, most of them also lacked confidence in the effectiveness of this solution or even feared retaliation:

“*You want to go to the police so people know what is happening at school and hopefully parents will try to protect their children. But then maybe the police will say ‘how do you know of these things, little girl, did you provoke this man?’ Maybe the school will pay the police to do nothing. And then people know what happened to you and they will judge you. So, it’s better to stay silent*.” [Girl FGD-F-16-057]

“*If a boy or a girl complains, there can also be negative consequence that might happen to them because if the problem involves the teacher, he might make you repeat the class, you’ll become like his enemy and the enemy of other teachers, and they will hold a grudge against you and harm you in class*.” [Boy FGD-M-16-085]

##### Internalisation of responsibility and behavioural self-regulation

Furthermore, the perceived legitimacy of certain acts of violence placed the onus of changing one’s behaviour on the victim:

“*If a teacher beats a boy because he’s disruptive, he must accept what happened because he was not obedient. So, he must first behave well and learn to interact with others*.” [Boy FGD-M-15-087]

“*If a girl experiences sexual violence, she must avoid playing too many games, because this often happens during recess if the girl plays too much, some boys will take advantage of the situation to touch their private parts. […] This kind of violence does not happen to girls who make themselves small and no one notices them*.” [Girl FGD-F-17-078]

Echoing the quote above, not standing out, not attracting attention to oneself, be it through clothing or attitude, was the most often quoted indication of a student “being smart” according to their peers. Correspondingly, when asked to define a “good student”, nearly all respondents prioritised behavioural traits over academic performance. A good student was almost universally described as “*polite*,” “*respectful*,” “*quiet*,” “*on time*,” and “*not disruptive*.” When grades or application to their homework were mentioned, it always came after those first qualities.

“*When we say that this girl is a good student, we mean that she is a girl who behaves well in class, behaving well in class means that she is a girl who always comes to school on time, a girl who does not disturb in class, a girl who respects the laws or the rules of the school, a girl who also defends the values of her family: she does not argue in class, she is also clean and she respects herself*.” [Girl IDI-F-16-021]

##### Perceived unfairness of gendered asymmetries in available coping strategies

The participants, however, pointed to a strongly gendered difference in the range of strategies available to navigate everyday school violence. All FGDs and interviews, regardless of participants’ gender, outlined a situation they considered somewhat unfair: girls can leverage harassment to their advantage, while boys have no other option but to escalate violence.

“*If a boy does not know the answer and the teacher tells him he’s an idiot, the boy can only insult the teacher back, maybe yell or hit him. Some boys get angry because that’s all they can do. But a girl can always go see the teacher after the class and say: ‘if you give me a good grade, I will go out with you’. It’s not really fair*.” [Boy FGD-M-15-090]

This pattern is perceived as continuing into adulthood, where men “*can only work very hard all the time*” when women “*always have the option of giving their bodies for a promotion, a better life*”. This ever-present possibility of using transactional sex for security or advancement was perceived by both boys and girls groups as a key gendered injustice.

“*Boys and girls have different opportunities. A boy will have no choice but to work very hard to support his family, he must even beg for jobs. Whereas a girl, she can marry someone already installed and she will be taken care of. She can even get a job if she’s not smart, only if she’s pretty and the boss likes having a pretty girl*.” [Girl IDI-F-15-006]

With this perspective, students oscillated between recognising that a teacher asking for sexual favours in exchange for grades might be abusive but were unsure whether girls who sought to capitalise on their body should be fully identified as victims.

“*If a boy fails in class, the teacher can ask him for money, but how is he going to find money? Whereas a girl, she always has her body she can give. Even if she does not want to go out with the teacher in the beginning of the year, if she sees she has bad grades, she can always put on a low-cut shirt, and she will not fail. She will not respect herself, but she will not fail*.” [Boy FGD-M-15-089]

In this quote, playing along and leveraging harassment to one’s advantage appears as a possible strategy, albeit one that might attract negative judgment from one’s peers.

##### Moral standing, credibility, and the threat of compounded reputational harm

In addition to contributing to legitimising certain instances of violence, the weight of reputation within the community also emerged as a powerful motivation to remain silent, along with the perception of the profoundly arbitrary nature of life in the DRC. The risk of “double punishment”, experiencing violence and subsequently suffering reputational damage, made reporting abuse a precarious decision for many students. This was compounded by the fact that community judgment appeared largely predetermined by an individual's existing reputation. If someone was perceived as respectful and well-behaved, the community was more likely to support them in the face of violence. Conversely, individuals seen as defiant or disrespectful were often blamed for their own victimisation, with acts of violence against them perceived as justified or even inevitable. This dynamic reinforced a culture of silence, where survivors hesitated to speak out for fear of being judged rather than supported.

“*For the adults at school, it depends on your reputation, if you are a good, polite, intelligent person and others they can stand by you and ask the management to review your case, but if you are rude, they can also condemn you by saying that it is your fault and you must take full responsibility. Even the people in the community too, it depends on how you behave with them, if you’re known as someone who is polite and well-behaved, they will be sorry. But if they say you had a bad behavior before, then everyone will agree you had it coming*.” [Boy FGD-M-17-081]

“*Yes, if a boy or a girl complains, there can be negative consequences if people already know you as someone who’s always at fault. Even when you’re right and you go to report it, they’ll say, ‘You’ve always been like that.’ The problem turns against you because of your usual bad behavior. So, the student tells themselves, even if I go report it, it won’t help—so I just stay silent because it might turn against me*.” [Girl IDI-F-16-003]

This was further exacerbated by the deeply entrenched social structures in the Democratic Republic of Congo where community reputation tends to supersede individual rights. Within such a framework, maintaining one's social standing is often considered more critical than seeking justice or protection from harm. The fear of “losing face” or being publicly shamed acted as a powerful deterrent against reporting violence. Many students internalised this norm, advising their peers to remain silent to avoid social ostracisation. As one participant summarised: “*Forget it because that will give you a bad reputation*”, highlighting the weight of reputational concerns in shaping responses to violence.

## Discussion

This study offers critical insights into how adolescents living in Kinshasa experience, define, and navigate school-based gender-based violence. Its findings show that violence is not only pervasive and multifaceted, but also deeply relational and embedded in broader socio-cultural and institutional frameworks. Echoing views from the existing literature, the adolescents’ lived experiences analysed here illustrate how violence is not simply a set of individual acts but is intrinsically shaped by broader sociological forces framing and defining the experience itself.^22,24^ Participants systematically situated violence within a web of social relationships and institutional expectations, underscoring how meaning, legitimacy, and responsibility are collectively produced rather than individually defined. This led to what researchers would classify as objectively violent acts (slapping, humiliating or fondling) being perceived as legitimate if perpetrated in response to a breach of accepted boundaries or social, and particularly gendered, roles. Students often rationalised punitive actions as educational, particularly when inflicted on boys who were almost always described as “naturally” unruly and thus logical perpetrators and recipients of violence. This echoes similar findings from other sub-Saharan African countries where physical and emotional violence were considered valid and even necessary forms of discipline.^25^ It also confirms conclusions from an earlier quantitative study in a similar setting, where out of a sample of 309 students, 74.4% agreed that it was good for a teacher to slap or beat a student if they disturb the class.^21^

Boys also presented aggression and escalation as the only response possible to assert dignity and regain power, suggesting the internalisation of gender norms where violence is a core expectation of masculinity, a pattern also found in ethnographies of school gender dynamics in Uganda.^26^ This limitation of boys’ available strategies might also indirectly sustain violence against girls by normalising violence, entitlement, and hetero-patriarchal norms. The legitimisation of violence perpetuated on girls was framed by strongly similar gendered expectations, where adolescents who deviated from expected behaviour norms (most often through their clothing or attitudes) almost systematically lost the status of victim. This assessment was conversely supported by the unanimous condemnation of any arbitrary punishments or sexual harassment perpetrated on students (boys and girls) who “had done nothing” to stand out.

Of all the forms of violence experienced by participants, the pervasive exchange of sexual favour for grades, a practice widely reported in higher education institutions in sub-Saharan Africa,^27–29^ but rarely documented in high schools, perhaps best illustrates the ambiguous and socially pre-informed interpretations of what constitute violence for adolescents in Kinshasa. While they theoretically understood the difference between consensual and coercive relationships, both boys and girls did not consistently condemn either the behaviours or the perpetrators. The normalising of men’s sexual entitlement not only placed a disproportionate burden of responsibilities on girls, but also occasionally flipped the perception of what constituted an injustice: boys viewed hard work and academic merit as their only legitimate pathway to success, while girls were seen (and saw themselves) as having the strategic option to “leverage their bodies” when all else failed. In this perspective, the option to be sexually exploited was paradoxically presented as an unfair advantage for girls and later women. The fact that girls themselves internalised being sexually exploited as an available strategic option underscores the pervasiveness of this narrative. Within such framing, objective gender and power imbalances and abusive practices are perverted and interpreted as a form of agency, thus shifting blame onto victims themselves, a dynamic frequently noted in the emerging literature on sexual corruption and sextortion.^30,31^ From a rights-based perspective, this reframing is particularly concerning, as it obscures violations of adolescents’ rights to bodily autonomy, dignity, and education by recasting coercion as choice and exploitation as strategy. Such narratives normalise abuse while eroding educational institutions’ obligations to protect students from harm and to ensure safe, equitable learning environments, as emphasised in rights-based approaches to adolescent health and education.

Our phenomenological analysis further showed how these structural discourses are translated into adolescents’ everyday lives through internalised processes of social judgment, and how the community gaze is an integral component of the lived experience of violence itself. This appeared not only in the stronger reaction of students confronted with any form of public humiliation, especially if it involved economic burden that may be passed on to poorer parents, but also in their description of the potential reactions to their denouncing acts of violence perpetrated against them. All our findings agree that victims are never evaluated solely on the basis of the act committed against them, but are “pre-judged” through the lens of their past behaviour and reputation. Within such a framework, even clearly abusive behaviour can be rationalised or overlooked if the victim is not seen as “respectable.”

As social norms and community perceptions are deeply embedded in adolescents’ lived experiences, they shape not only how violence is anticipated and endured, but also often silently accommodated by adolescents themselves. As a result, social expectations and (gendered) reputations not only outweigh individual rights but also affect available justice mechanisms. Students almost unanimously described extremely narrow options for them to address school-based violence. Reporting abuse was viewed as futile or even dangerous, particularly when teachers or school leadership were seen as protective of one another. Confirming findings from other studies conducted in sub-Saharan Africa,^32–34^ the risk of retaliation, along with the often greater fear of reputational harm, emerged as key reasons why students consistently prioritised remaining silent and trying to conform to expectations (being quiet, respectful, and appropriately dressed) as their main defence strategies against violence. Such injunctions (particularly those directing girls to “make themselves small” and avoid visibility) effectively constrain their participation in the classroom and undermine their ability to fully exercise their right to education, as silence and self-effacement become conditions for safety rather than learning.

Finally, the high levels of poverty pervasive in Kinshasa compounded several of these risks of both exposure to violence and limited options to navigate it for high school students. Tuition debts and fear that failing and repeating classes would lead to further economic burden could push them to comply with exploitative demands. This finding illustrates the added value of the phenomenological approach used in this study; economic coercion, rarely captured in studies of school-based violence relying on pre-defined survey categories,^30^ emerged strongly as a subtheme when adolescents described harmful situations in their own terms. While available studies to date mainly focuses on misappropriation of school resources and demands placed on parents,^35,36^ additional research appears needed to highlight economic coercion directly placed on students and its impact on them, particularly in high scarcity environments.

Overall, the multilayered dimensions of violence experienced by students in Kinshasa’s high schools align with scholarship emphasising that school-based violence must be understood as embedded within broader social ecologies, where community norms, gender regimes, and reputational logics shape both the interpretation of harm and the boundaries of acceptable behaviour. Prior studies similarly show that adolescents’ experiences of violence are coproduced through interactions between institutional authority, peer dynamics, and community-level expectations.^23,26,37–39^

Considering the weight of the community and social networks in the interpretation of the everyday instances of violence, but also in adolescents’ construction and perceptions as individuals, any meaningful harm-reduction intervention must therefore extend beyond school settings. It should address the internalised social norms that shape behavioural expectations, and legitimise or dismiss “violence”, while explicitly reaffirming adolescents’ rights to protection, dignity, and education within both their schools and their surrounding communities.

Our findings therefore add to the argument made elsewhere that interventions focusing solely on individual knowledge, curriculum changes, or formal prohibition are unlikely to succeed when harmful practices are embedded in widely shared beliefs about what is “normal,” “deserved,” or “inevitable”.^23,38,40^ In a scoping review of more than 100 articles on GBV in sub-Saharan Africa, Ezenwosu and Uzochukwo highlighted how interventions engaging community leaders and key influencers to change their beliefs about harmful social norms are among the most effective to sustainably reduce violence, although they also noted the dearth of research on impactful interventions in school settings.^41^ Results from the phenomenological analysis presented here further support the need for integrated, multi-level interventions that treat schools not as insulated or inherently protective spaces, but as sites where broader social hierarchies and gendered norms are reproduced but might also be negotiated and contested.

Several procedures were implemented to enhance the credibility and validity of these findings. First, the use of both FGDs and IDIs enabled triangulation across data collection methods. Second, data collection tools were iteratively refined through team training, pre-testing, and collective review. Third, the phenomenological approach deliberately avoided pre-defined categorisations, allowing adolescents to define violence in their own terms, and finally, independent coding by multiple researchers followed by consensus-based reconciliation increased analytic rigour and interpretive reliability.

Despite these methodological efforts, some limitations to this research should be acknowledged. First, the study is based on a purposive sample of students from only four schools in Kinshasa. While participant characteristics such as age, gender, school type, and prior exposure to school-based violence may have shaped how violence was experienced and interpreted, particularly with respect to norms around discipline, gender roles, and reputation, the strong consistency of accounts across FGDs and IDIs suggests that the patterns identified reflect shared dynamics within urban secondary schools in Kinshasa. These findings may, however, not be directly transferable to more rural educational contexts, where social norms, school governance, and community dynamics may differ.

Second, the combination of focus groups and individual interviews may have produced variations in students’ narratives, with group dynamics influencing the depth and nature of disclosures. Students’ responses may also have been influenced by social desirability bias, especially in focus groups, despite efforts to protect confidentiality and ensure gender-matched facilitation. As noted above, however, the strong thematic consistency observed across data collection methods supports the robustness of the core patterns identified.in consequence.

Third, participants were self-selected, which may have introduced a bias towards students who had more important or specific experience of school-based GBV they wanted to share. But as a consequence, these students may also have been able to provide richer responses and interpretations, which is consistent with the study’s objective of examining how adolescents narrate and make sense of harmful situations.

Fourth, while the research team made concerted efforts to preserve the original meanings during the translation of transcripts from Lingala to French, and later into English for publication, some nuance may have been lost, especially as only one author had the necessary linguistic skills to translate the illustrative quotes presented here from French to English. Concepts like “violence,” “respect,” and “normalcy” are culturally and linguistically specific, and even careful translation cannot fully capture embedded meanings. Additional research could incorporate participant validation or collaborative translation processes to further strengthen the cultural and linguistic fidelity of translated materials.

Finally, while the study explored students’ perceptions and experiences in depth, it did not include the perspectives of teachers, parents, or school administrators, which could provide a more holistic view of school-based GBV dynamics and highlight opportunities for interventions. Given the stated importance of designing community-wide interventions, it will be crucial for future research to include these voices.

## Conclusion

This study reveals that gender-based violence in schools in Kinshasa is pervasive, institutionally embedded, and interpreted through a lens shaped by gendered norms, social expectations, and economic vulnerability. Students interpret and respond to violence through a framework that privileges conformity, reputation, and silence over disclosure and justice. Gendered expectations compound these pressures, leaving girls particularly vulnerable to loss of bodily autonomy and dignity. But boys also appear strongly constrained by those expectations, which give them limited opportunities for alternative coping mechanisms and solidarity-building strategies. Overall, the educational system, instead of being a protective and empowering space, seems to replay along the same fault lines of social and gender inequalities coursing through Congolese communities.

In a context where reputational logics outweigh individual rights and abuse is rationalised by community standards, school-based GBV interventions should necessarily address the larger institutional and structural drivers (both norms and groups) of everyday violence, in order to safeguard adolescents’ rights, ensure equitable access to education and support the country’s investments towards broader development goals.

## Supplementary Material

Supplemental Table 2. Participant Characteristics

Supplemental Table 1. Key themes and subthemes

## Footnotes

* Officially, the legislation in DRC protects children’s rights from all forms of physical and psychological violence, including abuse committed by those in position of authority (teachers, school staff) per Law n° 09/001 of 10 January 2009, on the Protection of the Child. The DRC is also a signatory to multiple international conventions (UN Convention on the Rights of the Child, African Charter on the Rights and Welfare of the Child) explicitly prohibiting corporal punishments. In its guidelines, the Ministry of Education formally discourages violent disciplinary practices and all forms of economic or sexual exploitation by school personnel.
