# Supplementary material for: “What bothers the heart”: a phenomenological study of adolescents’ experiences and interpretations of school-based gender-based violence in Kinshasa, DRC
Source: Sex Reprod Health Matters. 2026 Jul 13;33(1):2702756. doi: 10.1080/26410397.2026.2702756 (PMC13455695; doi:10.1080/26410397.2026.2702756)
Supplement: Supplementary file 1 [file ZRHM_A_2702756_SM5883.docx]

Supplemental Table 2. Participant characteristics

| **Unique ID** | **FGD or IDI** | **Gender** | **Age** | **PROVED** | **School** |
| --- | --- | --- | --- | --- | --- |
| IDI-F-16-001 | IDI | Female | 16 | Funa | Public |
| IDI-F-15-002 | IDI | Female | 15 | Funa | Public |
| IDI-F-16-003 | IDI | Female | 16 | Funa | Private |
| IDI-F-16-004 | IDI | Female | 16 | Funa | Private |
| IDI-F-17-005 | IDI | Female | 17 | Funa | Religious - Protestant |
| IDI-F-15-006 | IDI | Female | 15 | Funa | Religious - Protestant |
| IDI-F-16-007 | IDI | Female | 16 | Funa | Religious - Catholic |
| IDI-F-17-008 | IDI | Female | 17 | Funa | Religious - Catholic |
| IDI-F-15-009 | IDI | Female | 15 | Lukunga | Public |
| IDI-F-15-010 | IDI | Female | 15 | Lukunga | Public |
| IDI-F-16-011 | IDI | Female | 16 | Lukunga | Private |
| IDI-F-17-012 | IDI | Female | 17 | Lukunga | Private |
| IDI-F-15-013 | IDI | Female | 15 | Lukunga | Religious - Protestant |
| IDI-F-16-014 | IDI | Female | 16 | Lukunga | Religious - Protestant |
| IDI-F-16-015 | IDI | Female | 16 | Lukunga | Religious - Catholic |
| IDI-F-17-016 | IDI | Female | 17 | Lukunga | Religious - Catholic |
| IDI-F-16-017 | IDI | Female | 16 | Mont Amba | Public |
| IDI-F-15-018 | IDI | Female | 15 | Mont Amba | Public |
| IDI-F-16-019 | IDI | Female | 16 | Mont Amba | Private |
| IDI-F-15-020 | IDI | Female | 15 | Mont Amba | Private |
| IDI-F-16-021 | IDI | Female | 16 | Mont Amba | Religious - Protestant |
| IDI-F-15-022 | IDI | Female | 15 | Mont Amba | Religious - Protestant |
| IDI-F-15-023 | IDI | Female | 15 | Mont Amba | Religious - Catholic |
| IDI-F-17-024 | IDI | Female | 17 | Mont Amba | Religious - Catholic |
| IDI-F-16-025 | IDI | Female | 16 | Tshangu | Public |
| IDI-F-15-026 | IDI | Female | 15 | Tshangu | Public |
| IDI-F-15-027 | IDI | Female | 15 | Tshangu | Public |
| IDI-F-15-028 | IDI | Female | 15 | Tshangu | Private |
| IDI-F-15-029 | IDI | Female | 15 | Tshangu | Private |
| IDI-F-15-030 | IDI | Female | 15 | Tshangu | Private |
| IDI-F-16-031 | IDI | Female | 16 | Tshangu | Religious - Protestant |
| IDI-F-16-032 | IDI | Female | 16 | Tshangu | Religious - Protestant |
| IDI-F-16-033 | IDI | Female | 16 | Tshangu | Religious - Protestant |
| IDI-F-16-034 | IDI | Female | 16 | Tshangu | Religious - Catholic |
| IDI-F-15-035 | IDI | Female | 15 | Tshangu | Religious - Catholic |
| IDI-F-15-036 | IDI | Female | 15 | Tshangu | Religious - Catholic |
| IDI-M-17-037 | IDI | Male | 17 | Funa | Public |
| IDI-M-16-038 | IDI | Male | 16 | Funa | Private |
| IDI-M-16-039 | IDI | Male | 16 | Funa | Religious - Protestant |
| IDI-M-16-040 | IDI | Male | 16 | Funa | Religious - Catholic |
| IDI-M-16-041 | IDI | Male | 16 | Lukunga | Public |
| IDI-M-16-042 | IDI | Male | 16 | Lukunga | Private |
| IDI-M-15-043 | IDI | Male | 15 | Lukunga | Religious - Protestant |
| IDI-M-16-044 | IDI | Male | 16 | Lukunga | Religious - Catholic |
| IDI-M-17-045 | IDI | Male | 17 | Mont Amba | Public |
| IDI-M-16-046 | IDI | Male | 16 | Mont Amba | Private |
| IDI-M-15-047 | IDI | Male | 15 | Mont Amba | Religious - Protestant |
| IDI-M-16-048 | IDI | Male | 16 | Mont Amba | Religious - Catholic |
| IDI-M-17-049 | IDI | Male | 17 | Tshangu | Public |
| IDI-M-15-050 | IDI | Male | 15 | Tshangu | Private |
| IDI-M-15-051 | IDI | Male | 15 | Tshangu | Religious - Protestant |
| IDI-M-15-052 | IDI | Male | 15 | Tshangu | Religious - Protestant |
| IDI-M-17-053 | IDI | Male | 17 | Tshangu | Religious - Catholic |
| IDI-M-15-054 | IDI | Male | 15 | Tshangu | Religious - Catholic |
| FGD-F-17-055 | FGD | Female | 17 | Funa | Religious - Catholic |
| FGD-F-16-056 | FGD | Female | 16 | Funa | Religious - Catholic |
| FGD-F-16-057 | FGD | Female | 16 | Funa | Religious - Catholic |
| FGD-F-17-058 | FGD | Female | 17 | Funa | Religious - Catholic |
| FGD-F-17-059 | FGD | Female | 17 | Funa | Religious - Catholic |
| FGD-F-15-060 | FGD | Female | 15 | Funa | Religious - Catholic |
| FGD-F-15-061 | FGD | Female | 15 | Lukunga | Religious - Protestant |
| FGD-F-16-062 | FGD | Female | 16 | Lukunga | Religious - Protestant |
| FGD-F-17-063 | FGD | Female | 17 | Lukunga | Religious - Protestant |
| FGD-F-15-064 | FGD | Female | 15 | Lukunga | Religious - Protestant |
| FGD-F-15-065 | FGD | Female | 15 | Lukunga | Religious - Protestant |
| FGD-F-15-066 | FGD | Female | 15 | Lukunga | Religious - Protestant |
| FGD-F-15-067 | FGD | Female | 15 | Mont Amba | Public |
| FGD-F-15-068 | FGD | Female | 15 | Mont Amba | Public |
| FGD-F-16-069 | FGD | Female | 16 | Mont Amba | Public |
| FGD-F-15-070 | FGD | Female | 15 | Mont Amba | Public |
| FGD-F-15-071 | FGD | Female | 15 | Mont Amba | Public |
| FGD-F-17-072 | FGD | Female | 17 | Mont Amba | Public |
| FGD-F-16-073 | FGD | Female | 16 | Tshangu | Private |
| FGD-F-15-074 | FGD | Female | 15 | Tshangu | Private |
| FGD-F-17-075 | FGD | Female | 17 | Tshangu | Private |
| FGD-F-16-076 | FGD | Female | 16 | Tshangu | Private |
| FGD-F-17-077 | FGD | Female | 17 | Tshangu | Private |
| FGD-F-17-078 | FGD | Female | 17 | Tshangu | Private |
| FGD-M-15-079 | FGD | Male | 15 | Lukunga | Religious - Catholic |
| FGD-M-17-080 | FGD | Male | 17 | Lukunga | Religious - Catholic |
| FGD-M-17-081 | FGD | Male | 17 | Lukunga | Religious - Catholic |
| FGD-M-15-082 | FGD | Male | 15 | Lukunga | Religious - Catholic |
| FGD-M-16-083 | FGD | Male | 16 | Lukunga | Religious - Catholic |
| FGD-M-17-084 | FGD | Male | 17 | Lukunga | Religious - Catholic |
| FGD-M-16-085 | FGD | Male | 16 | Mont Amba | Religious - Protestant |
| FGD-M-16-086 | FGD | Male | 16 | Mont Amba | Religious - Protestant |
| FGD-M-15-087 | FGD | Male | 15 | Mont Amba | Religious - Protestant |
| FGD-M-15-088 | FGD | Male | 15 | Mont Amba | Religious - Protestant |
| FGD-M-15-089 | FGD | Male | 15 | Mont Amba | Religious - Protestant |
| FGD-M-15-090 | FGD | Male | 15 | Mont Amba | Religious - Protestant |
