# Supplementary material for: “What bothers the heart”: a phenomenological study of adolescents’ experiences and interpretations of school-based gender-based violence in Kinshasa, DRC
Source: Sex Reprod Health Matters. 2026 Jul 13;33(1):2702756. doi: 10.1080/26410397.2026.2702756 (PMC13455695; doi:10.1080/26410397.2026.2702756)
Supplement: Supplementary file 2 [file ZRHM_A_2702756_SM8612.docx]

| **Key themes** | **Subthemes** | ***Illustrative quotes*** |
| --- | --- | --- |
| **Multifaceted forms of violence in schools** | Inter-student violence and fights | *Fights between students happen all the time because sometimes we joke and somebody gets offended and then there is a fight. Sometimes you insult someone and they directly slap you. Even the girls pull each other hairs over some arguments.* |
|  | Corporal punishments from teachers | *Once in computer class, the teacher turned around and slapped me, and I didn’t even know why. He said, “you were talking too much” but I said I was not and then he slapped me again and said, “that’s because your shirt is not buttoned correctly”. I’m always afraid of that teacher.* |
|  | Emotional violence and humiliations | *The teachers giving you discipline is one thing but often they insult you, if you don’t know the answer, instead of explaining or encouraging you, they say “you’re stupid, you’ll be unemployed like your parents, you’re like dogs I cannot teach you anything.” And when they say that in front of your classmates, it breaks your heart and then you cannot concentrate anymore.* |
|  | Economic violence | *The teacher will tear your notebook because your writing is poor, and it means your parents have to buy a new one and that gets you in trouble at home too.   Sometimes you do nothing wrong, and a teacher searches your bag and confiscates your phone and then you have to pay him XOF 5,000 [about $1.8] to get it back, they don’t even care if your family is poor, it’s very unfair.* |
|  | Sexual violence: touching, fondling, online and verbal harassment | *There’s a [male] teacher who always says, “the first one I catch cheating in the exam, I will punish them and if it’s a girl, I’ll kiss her and I’ll squeeze her breasts.”   Once on WhatsApp I see a number I don’t recognize, and someone says “Hi” and then “Send me nudes!” And I realized it was our Head Teacher, and I didn’t know what to do. I received so many messages from him asking for sexy pictures. But then at school I told him my older sister had seen the messages, and he was very embarrassed, and he stopped.* |
|  | Sexual extortion and sex-for-grades | *It happens very often that a teacher falls in love with a student, and if the [girl] refuses, he will openly say «then you will fail my class » and the girl will have to have sex with him. It’s already forcing her to have sex because of the grade in the class. This is really not good.  Sometimes even female teachers, they will ask boys to stay after class because they want to kiss them and touch them. And they will promise extra points in the class if the boy lets them do what they want and even boys are not always comfortable with that.   With teachers this sometimes happens when you have difficulty understanding certain lessons, then the teacher can ask you to have sex with him so that he gives you the points, if the girl is weak and she is also afraid to repeat the class, she can also accept the teacher's request to have sex with him […] Sometimes you can also be smart in his class but when he calls you into their office to buy him things (water, juice etc.) and if you don’t have the money, he will also take advantage to ask you to go out with him.* |
| Legitimized forms of violence | Physical violence as necessary discipline | *The fact that some students protest against the teachers' decisions, if a teacher hits him, and then the student continues to protest or hits his teacher back, this is what increases the case of physical violence at school. I personally find that it is normal for a teacher to whip a student as part of educating him or to discipline him to teach a lesson to other students who are disturbing the class.* |
|  | Breach of gendered expectations | *There are other girls in our school who want to be seen at all times, girls who come to school only to look for boys, they want to be noticed by everyone at school at all costs, they wear short skirts, tight clothes, these kinds of girls if they suffer from violence, I think it's their fault.  When we say that this girl is a good student, we mean that she is a girl who behaves well in class, behaving well in class means that she is a girl who always comes to school on time, a girl who does not disturb in class, a girl who respects the laws or the rules of the school, a girl who also defends the values of her family: she does not argue in class, she is also clean and she respects herself.* |
|  | Victim-blaming dynamics | *It can be her fault if she doesn’t respect herself, because there are also boys who are afraid to approach girls who respect themselves, but those who don’t respect themselves experience these kinds of trouble* |
|  | Male sexual urges cannot be controlled | *The agents of the Ministry of Public Health told us that it is not at all good to wear transparent clothes if we want to prevent men/boys from falling into sexual violence [...] I know a man who raped a girl because of her clothing [That is why] I would ask girls to avoid clothes that are too sexy because it also attracts men/boys and forces them to have negative thoughts about them, or to have sexual desires at all costs* |
|  | Understanding of consent | *I would say yes, sexual violence can happen to people who are also in a relationship, this is the case in our relationships with our boyfriends, it sometimes happens that the man starts to force you to make love even if you don't want to do it, this is already sexual violence* |
| Unacceptable practices | Disproportionate punishments | *We have this guardian, he's truly rough, he always waits by the school entrance and even if it rains he makes you remove your jacket before getting into the school to check your uniform. If you wear sneakers that are not white like the school policies say, then he slaps you or make you go back home and then slaps you again when you're late. This is really not fair.* |
|  | Abitrary targeting (hurting students who "have done nothing") | *It’s the worst thing that can happen is when you’re sitting quietly and you’re not insolent, but then a teacher falls in love with you, and they want to touch you.* |
|  | Betraying duty of care | *When you’re at school, you’re still a child, you’re not dressing like a woman, and if a teacher wants to go out with you, it’s wrong. Not just because you’re 15 and maybe they’re 50, but because they should be like a father to you. And that’s sexual violence* |
| **Coping mechanisms** | Remaining silent / not reporting violence | *I would tell them to remain silent or pretend not to hear anything in such situations […] they cannot tell this to their friends because [the friends] can also betray them by going to tell everything to the perpetrator of this violence and this can spread to the whole school, which is not good at all either.* |
|  | Ignoring trauma | *Some advice I would give to this person who has suffered this kind of violence is, do not think too much about it, because it will always make you feel guilty, what is past is already past, you just have to look forward.* |
|  | Conforming to norms and gender expectations | *If a teacher beats a boy because he’s disruptive, he must accept what happened because he was not obedient. So, he must first behave well and learn to interact with others.   If a girl experiences sexual violence, she must avoid playing too many games, because this often happens during recess if the girl plays too much, some boys will take advantage of the situation to touch their private parts. […] This kind of violence does not happen to girls who make themselves small and no one notices them* |
|  | Being sexually exploited as an unfair option for women | *If a boy does not know the answer and the teacher tells him he’s an idiot, the boy can only insult the teacher back, maybe yell or hit him. Some boys get angry because that’s all they can do. But a girl can always go see the teacher after the class and say: “if you give me a good grade, I will go out with you”. It’s not really fair.  Boys and girls have different opportunities. A boy will have no choice but to work very hard to support his family, he must even beg for jobs. Whereas a girl, she can marry someone already installed and she will be taken care of. She can even get a job if she’s not smart, only if she’s pretty and the boss likes having a pretty girl.  If a boy fails in class, the teacher can ask him for money, but how is he going to find money? Whereas a girl, she always has her body she can give. Even if she does not want to go out with the teacher in the beginning of the year, if she sees she has bad grades, she can always put on a low-cut shirt, and she will not fail. She will not respect herself, but she will not fail.* |
| **Social forces at work** | Existing reputation mediates credibility and blame | *It depends on your behavior before […] if people know you’re well-behaved, if you dress correctly, if you’re smart and people harass you, then they will blame the perpetrator of the violence and not you.  For people in the community if you are an insolent child, they may say that it is normal that negative consequences happen to you, but if you are an intelligent, serious child who also respects people, they will feel sorry for you.  For the adults at school, it depends on your reputation, if you are a good, polite, intelligent person and others they can stand by you and ask the management to review your case, but if you are rude, they can also condemn you by saying that it is your fault and you must take full responsibility. Even the people in the community too, it depends on how you behave with them, if you’re known as someone who is polite and well-behaved, they will be sorry. But if they say you had a bad behavior before, then everyone will agree you had it coming* |
|  | Mistrust in institutions | *You want to go to the police so people know what is happening at school and hopefully parents will try to protect their children. But then maybe the police will say “how do you know of these things, little girl, did you provoke this man?”. Maybe the school will pay the police to do nothing. And then people know what happened to you and they will judge you. So, it’s better to stay silent.* |
|  | School personnel and teacher collusion | *Most of the time, these are situations where you can't say anything or do anything, this is the case of a teacher who wants to go out with me at all costs, who bothers me at any time and asks for my phone number, as a student I don't want to say anything or I don't want to report it, because sometimes when you report such a case to the management and the teacher denies everything, due to lack of evidence the student can be permanently excluded from the school. As we say “wolves don’t eat each other” so the director is not going to be on my side. That’s why I can't do anything or react; I just want to let this problem go.* |
|  | Retaliation risk | *If a boy or a girl complains, there can also be negative consequence that might happen to them because if the problem involves the teacher, he might make you repeat the class, you’ll become like his enemy and the enemy of other teachers, and they will hold a grudge against you and harm you in class.* |
|  | Reputational risk ("double punishment") | *Yes, if a boy or a girl complains, there can be negative consequences if people already know you as someone who’s always at fault. Even when you’re right and you go to report it, they’ll say, ‘You’ve always been like that.’ The problem turns against you because of your usual bad behavior. So, the student tells themselves, even if I go report it, it won’t help—so I just stay silent because it might turn against me.* |
